# Health Insurance and Preventive Medicine

**Published:** 1912-07-27

**Authors:** 


### HEALTH INSURANCE AND PREVENTIVE MEDICINE.

When Milton made the agonising Samson bitterly voice the feeling of despair and dignified resignation, blent with a streak of honest anger at a fate to such a character more desperate than death, he managed to convey, in the use of one or two subtly chosen words, an essence of finality that heightens the impression of gloom. Sir Clifford Allbutt, far less potent a blender of language, is hampered even more by his choice of subject than by his inability to rival Milton's roll. In a letter which he forwarded to the *Times* this week he tilts against the Insurance Act. His tilting is dexterous, and if here and there it be marred by a scholastic attempt to pile Pelion on Ossa, and an inartistic flourish of supposititious similes, yet its primary intention is against the Act. Perhaps for that very reason it is to be regretted that he has not rigidly subedited his letter, and, above all, amended the paragraph in which specific charges against State apathy are grouped. We do not complain so much about the remarkable statement that "beri-beri depends upon the integrity of a microscopic film on the seed of rice"—an assertion which, as we pointed out some time ago, is entirely unproved—but we hazard the hope that in future communications to the public press the Regius Professor of Medicine will give the State some credit for the advances in preventive medicine that have been made during the last ten years. Neither Chairs nor Professors have done so much to enlarge our knowledge of epidemic disease in this country as the men who are working on salaries paid by public bodies out of the rates.

We have shown in what we think he has argued unfairly; now let us examine the gravamen of his charge in the *Times* letter, which is that the Act may tend to stereotype a rule-of-thumb method of practice already, unfortunately, in being. That is a true and very grave danger. Those of us who believe with Professor Allbutt that the Act represents "a generous impulse and a generous idea," are convinced that it is at present an impulse which has been almost stifled by mismanagement. We may take courage and heart of grace by remembering the history of similar wide-ranging schemes elsewhere. Nothing exceeded the chaos consequent upon the passing of the Bavarian Sick Nursing Insurance Act of 1858, the prototype of all State sickness insurance schemes: to-day medicine nowhere in the world stands higher as a science than in Bavaria. We agree that the insurance system is not entirely responsible for that progress; public and State effort and private endeavour aided university and collegiate expansion and made possible a progress that has written Munich largely in the annals of medicine. But indirectly, as many authoritative writers have shown, German medicine owes much to the Bismarckian insurance system. The excellent arrangement whereby a German general practitioner is annually enabled to attend a course of graduate instruction classes—an arrangement quite recently praised by Professor Osler as being almost ideal—resulted from the general wish of the profession in Germany to do away with the rule-of-thumb method in diagnosis and treatment, and to substitute for it scientific methods based on the best modern teaching. We confess that we have not lost our hope that in this country too similar progress, and precisely similar effort, may follow the introduction of Mr. Lloyd George's Act. It is wholly true that the Act, as it now stands on the Statute-book, is a congenitally defective measure. But we venture to think that surgical treatment, in the shape of amendment, may yet do much to make it workable. Even in the direction whither Sir Clifford constantly directs his eyes—the establishment of a school of comparative medicine—it suggests an opening. By allocating a definite sum for reasearch and in the establishment of sanatoria it should yet supply another method of tackling preventive medicine on a grand scale. We do not like the astronomical similes introduced, but we sincerely trust that if "Copernican medicine" is an ideal to be striven for, the Act can yet help its attainment, if all of us who are members of the profession or who feel interested in the public welfare will strive to amend its provisions.

